# Predicting novel candidate human obesity genes and their site of action by systematic functional screening in *Drosophila*

**DOI:** 10.1371/journal.pbio.3001255

**Published:** 2021-11-08

**Authors:** Neha Agrawal, Katherine Lawler, Catherine M. Davidson, Julia M. Keogh, Robert Legg, INTERVAL, Inês Barroso, I. Sadaf Farooqi, Andrea H. Brand

**Affiliations:** 1 The Gurdon Institute and Department of Physiology, Development and Neuroscience, University of Cambridge, Cambridge, United Kingdom; 2 University of Cambridge Metabolic Research Laboratories and NIHR Cambridge Biomedical Research Centre, Wellcome-MRC Institute of Metabolic Science, Addenbrooke’s Hospital, Cambridge, United Kingdom; 3 MRC Epidemiology Unit, Institute of Metabolic Science, University of Cambridge, Cambridge, United Kingdom; The Francis Crick Institute, UNITED KINGDOM

## Abstract

The discovery of human obesity-associated genes can reveal new mechanisms to target for weight loss therapy. Genetic studies of obese individuals and the analysis of rare genetic variants can identify novel obesity-associated genes. However, establishing a functional relationship between these candidate genes and adiposity remains a significant challenge. We uncovered a large number of rare homozygous gene variants by exome sequencing of severely obese children, including those from consanguineous families. By assessing the function of these genes in vivo in *Drosophila*, we identified 4 genes, not previously linked to human obesity, that regulate adiposity (*itpr*, *dachsous*, *calpA*, and *sdk*). Dachsous is a transmembrane protein upstream of the Hippo signalling pathway. We found that 3 further members of the Hippo pathway, fat, four-jointed, and hippo, also regulate adiposity and that they act in neurons, rather than in adipose tissue (fat body). Screening Hippo pathway genes in larger human cohorts revealed rare variants in *TAOK2* associated with human obesity. Knockdown of *Drosophila tao* increased adiposity in vivo demonstrating the strength of our approach in predicting novel human obesity genes and signalling pathways and their site of action.

## Abstract 2

This study set out to identify novel gene variants that may contribute to human obesity, by combining human exosome sequencing analyses with systematic functional screening in Drosophila. This identifies a number of novel obesity-associated genes which control adiposity in flies, and uncovers a potential role for the Hippo signaling pathway in obesity.

Obesity is a major risk factor for type 2 diabetes, cardiovascular disease, cancers, and, most recently, COVID-19 [1]. Despite the obvious environmental drivers to weight gain, multiple genetic studies have demonstrated that 40% to 70% of the variation in body weight is attributable to genetic variation [2]. The discovery of genes that contribute to the regulation of human body weight can provide insights into the mechanisms involved in energy homeostasis and identify potential targets for weight loss therapy. Moreover, drug targets supported by human genetic evidence are more likely to transit successfully through the drug discovery pipeline [3].

A classical approach to the discovery of pathogenic variants is to investigate consanguineous populations with high degrees of parental relatedness (parents who are first or second cousins) where large portions of the genome are identical by descent as a result of family structure in preceding generations (long regions of homozygosity). Indeed, studies in consanguineous families led to the discovery of the first homozygous loss-of-function mutations in the genes encoding leptin (*LEP;* [4]) and the leptin receptor *(LEPR;* [5]) associated with severe obesity. However, at the time, the function of leptin and its receptor had already been established in *ob/ob* and *db/db* mice, respectively [6], so the pathogenicity of homozygous mutations that resulted in loss of function in cells was readily established.

The situation is more complex when studying homozygous mutations in new candidate genes. Some of these genes may play a direct causal role in the development of obesity, others may increase susceptibility to obesity only in certain contexts, and some genes will play no role at all. Recent large-scale studies in healthy people in outbred populations have revealed that a significant proportion of rare homozygous variants that are predicted to cause a loss of function do not result in a clinically discernible phenotype [7,8]. As such, identifying the subset of genes that may be involved in the regulation of adiposity in large human genetic studies presents a major hurdle.

For some diseases, functional screens in cultured cells permit rapid testing of candidate genes, as exemplified by studies of insulin secretion in islet cells for genes associated with type 2 diabetes [9]. However, obesity is a systems-level disorder that cannot be replicated in cells. As such, a functional screen in vivo is needed. Here, we use *Drosophila* to screen the functional consequences of knocking down expression of candidate human obesity genes and to explore the complex interactions between multiple organ systems that are regulated by environmental and genetic factors.

*Drosophila* has been a useful tool in the functional characterisation of human disease-associated genes [10–12]. Many organ systems and metabolic enzymes are highly conserved in *Drosophila*, as are the major regulatory mechanisms involved in metabolic homeostasis [13,14]. As in humans, *Drosophila* accumulate lipids and become obese when raised on a high-fat or high-sugar diet, developing cardiomyopathy and diabetic phenotypes [15,16]. Furthermore, more than 60% of the genes identified in an unbiased genome-wide RNAi screen for increased fat levels in *Drosophila* have human orthologues [17]. Most studies in *Drosophila* have performed forward genetic screens resulting in obesity [18] before assessing whether misregulation of the corresponding mammalian orthologue affects adiposity [17]. Another report knocked down *Drosophila* orthologs of human genes near body mass index (BMI) loci from GWAS studies to identify genes regulating adiposity [19].

Here, instead, we chose to take advantage of new data from a cohort of patients carrying rare genetic variants that might cause severe early-onset obesity. We set out to identify, in *Drosophila*, whether any of these genes are likely to be responsible for the obese phenotype. An additional advantage of working with *Drosophila* is the potential to identify interacting genes and signalling pathways. We proposed that it would then be possible to search for variants in human orthologues of these genes in larger cohorts of patients, to discover further as yet unidentified genes regulating human obesity.

To increase our chances of finding pathogenic variants, we focused on rare homozygous variants identified in probands with severe obesity, many from consanguineous families. After knocking down expression of *Drosophila* orthologues of candidate human obesity genes, we discovered 4 genes that significantly increased triacylglyceride (TAG) levels. Importantly, none of these genes had been associated previously with human obesity, but the pathways in which they act are known and could be further analysed in *Drosophila*. Knockdown of further members of one of these signalling pathways, the Hippo pathway, also gave an obesity phenotype, highlighting the success of our approach. We then searched for variants in the novel obesity genes we identified in *Drosophila*, and their associated signalling pathways, in larger cohorts of unrelated obese people and healthy controls. This uncovered yet another gene, which, when knocked down in *Drosophila*, increased adiposity. We demonstrate that the cross-fertilisation of human and *Drosophila* genetics is a powerful system to provide novel insights into the genetic and cellular processes regulating adiposity and may ultimately contribute to strategies for the prevention and treatment of obesity.

## Results

### Rare homozygous variants in individuals with severe early-onset obesity

**Fig 1:**
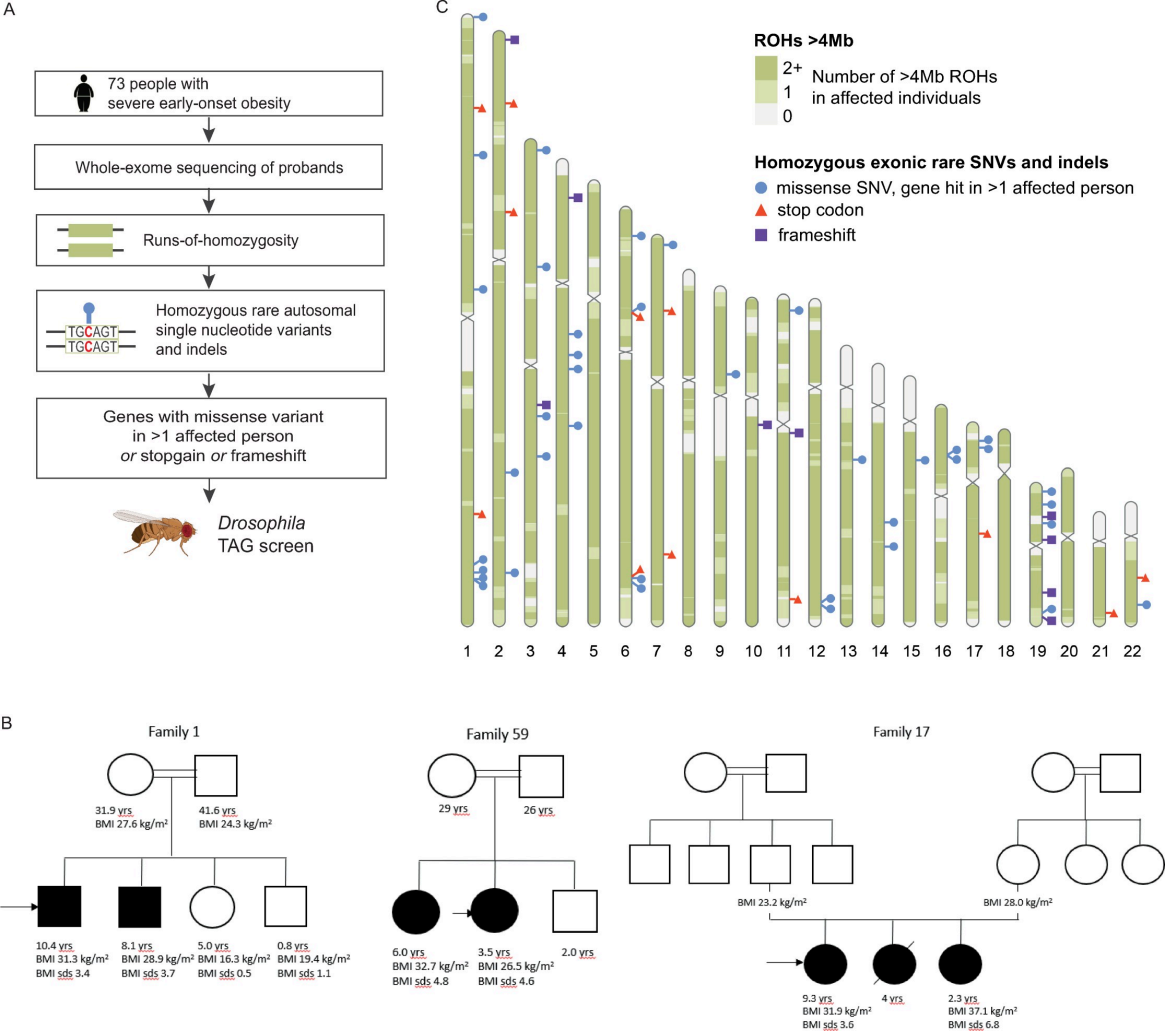
Identification of genes harbouring homozygous rare variants in severely obese individuals. (A) Schematic of the approach used to identify candidate human obesity genes and the prioritisation of genes containing rare exonic SNVs and indels for screening in *Drosophila*. (B) All probands had severe obesity and presented in early childhood, as exemplified in 3 family pedigrees shown. Males (squares) and females (circles) with obesity (filled symbols) are indicated. BMI in children under 18 years is adjusted for age and sex and shown as BMI SDS. Arrows indicate probands who were sequenced; slashed lines indicate deceased individuals; double lines indicate consanguineous union. Family numbers refer to **S1 Table**. (C) Cumulative medium to large ROHs in affected cases and prioritised homozygous exonic rare SNVs and indels. The autosomal karyotype heatmap illustrates the cumulative coverage of medium to large ROHs (>4 Mb) among the affected individuals, based on estimates of ROHs using WES data (displayed in bins of size 10^5^ bp). Markers show the location and consequence of homozygous rare exonic SNVs and indels within any ROHs (>100 kb). Stop codons and frameshifts are displayed, and missense variants where >1 affected person had a missense variant in the same gene. Variant markers are shown per gene **(S2 Table)**. BMI, body mass index; indel, insertion/deletion; ROH, run of homozygosity; SDS, standard deviation score; SNV, single-nucleotide variant; WES, whole-exome sequencing.

We performed whole-exome sequencing (WES) of 73 individuals with severe early-onset obesity recruited to the Genetics of Obesity Study (BMI standard deviation score [BMI SDS] > 3; age of onset below 10 years; GOOS; www.goos.org.uk), in whom known causes of monogenic obesity such as congenital leptin deficiency and *MC4R* deficiency had been excluded **(Fig 1A, S1 Table; Methods)**. Fifty-seven probands were offspring from consanguineous families in the United Kingdom (predominantly of South Asian origin; only probands were sequenced; **Fig 1B, S1 Table**). We looked for autosomal homozygous nonsynonymous single-nucleotide variants (SNVs) and insertions/deletions (indels) affecting exons or splice sites in affected individuals (**Methods**). On the assumption that homozygous variants that cause severe obesity are likely to be rare alleles, only variants with a minor allele frequency (MAF) <1% among all publicly available exomes (gnomAD) and <1% in the gnomAD South Asian subpopulation were retained.

We found a very large number of homozygous coding variants (*n* = 756) in regions of homozygosity >100 kb at 743 different genomic locations suggesting substantial genetic heterogeneity (**Fig 1C**). Among these were 12 nonsense variants and 9 frameshift variants **(Fig 1C, S2 Table)**. One proband harboured a homozygous rare nonsense variant in *ALMS1* consistent with a diagnosis of Alstrom’s syndrome, a disorder characterised by childhood obesity, visual impairment, hearing loss, and cardiomyopathy. To identify new genes for severe obesity, we focused on a set of 61 genes in which 1 person carried a frameshift or nonsense variant or at least 2 affected individuals carried a rare missense variant.

### Screening obesity candidate genes in *Drosophila melanogaster*

We identified *Drosophila* orthologues for each of the 61 genes, taking advantage of the *Drosophila* RNAi Screening Centre Integrative Ortholog Prediction Tool (DIOPT; Version 5) [20] (**S2 Table**). DIOPT calculates a score indicating the number of orthologue prediction tools that support a given orthologous gene-pair relationship. The *Drosophila* gene with the highest DIOPT score for each human gene was chosen for further study. Of the 61 genes in our study, DIOPT identified orthologues for 50 genes. Of these, 27 human genes with 24 *Drosophila* orthologues were selected (**Table 1**) based on the following criteria: high DIOPT scores; RNAi lines available from the *Drosophila* TRiP collection; and having been implicated previously in regulating metabolism. We excluded 6 *Drosophila* orthologues without preexisting RNAi lines and 15 genes with low DIOPT scores.

**Table 1:** *Drosophila* orthologues of candidate human obesity genes. The 24 *Drosophila* orthologues of 27 human genes with rare variants in obese patients, which were chosen for further study. The DIOPT score, TRiP RNAi line (except *itpr* (NIG line), and the function of each gene are given.

Human Symbol	Fly Symbol	DIOPT Score	TRiP line	Function
**ACO2**	**Acon**	**12**	**34028**	**Aconitase; conversion of citrate to isocitrate in TCA cycle**
**ACSM1**	**pdgy**	**1**	**55272**	**Acyl-CoA synthetase; activates FA destined for beta-oxidation**
**ACSM3**	**pdgy**	**1**	**55272**	**Acyl-CoA synthetase; activates FA destined for beta-oxidation**
**AGAP6**	**CenG1A**	**8**	**31228**	**Centaurin gamma 1A; GTPase; ecdysone signalling-dependent**
**ALK**	**Alk**	**12**	**27518**	**Anaplastic lymphoma kinase; development and growth**
**BMP2K**	**Nak**	**7**	**38326**	**Numb-associated kinase; dendrite development**
**CAPN8**	**CalpA**	**10**	**29455**	**Calcium-dependent endopeptidase; dorsal/ventral pattern**
**CDS1**	**CdsA**	**13**	**58118**	**CDP diglyceride synthetase; cell growth and lipid storage**
**CHIT1**	**Cht7**	**10**	**65000**	**Chitinase 7, chitin-based cuticle development**
**CPA4**	**CG3097**	**9**	**65948**	**Carboxypeptidase**
**DCHS1**	**ds**	**13**	**32964**	**Dachsous; cadherin; cell adhesion**
**DNAH10**	**Dhc98D**	**13**	**77181**	**Dynein Heavy Chain; minus end MT motors**
**ITPKB**	**IP3K2**	**10**	**55240**	**Kinase regulates calcium levels by influencing IP3 signaling**
**ITPR1**	**Itp-r83A**	**15**	**1063-R2 (NIG)**	**Inositol 1,4,5 trisphosphate receptor**
**LPIN1**	**Lpin**	**13**	**63614**	**Fat body function, downstream effector of insulin and TORC1**
**MYH15**	**Mhc**	**12**	**35729**	**Myosin Heavy Chain, motor protein for muscle contraction**
**NUP133**	**Nup133**	**13**	**58290**	**Nucleoporin; constitutent of nuclear pore complex**
**OVGP1**	**Cht7**	**7**	**65000**	**Chitinase 7; chitin-based cuticle development**
**PAPSS1**	**Papss**	**14**	**60471**	**Adenlylsulphate kinase; sulphate assimilation**
**PGBD4**	**CG9839**	**7**	**64933**	**PiggyBac transposable element-derived protein**
**PLEKHG1**	**GEFmeso**	**8**	**42545**	**Guanine nucleotide exchange factor in mesoderm**
**SCO1**	**Scox**	**14**	**55179**	**Synthesis of cytochrome C oxidase; copper chaperone**
**SDK1**	**sdk**	**8**	**33412**	**Sidekick, Fibronectin type; photoreceptor cell differentiation**
**SYNE1**	**Msp300**	**5**	**32377**	**Muscle-specific protein; positioning of muscle nuclei**
**SYNE2**	**Msp300**	**4**	**32377**	**Muscle-specific protein; positioning of muscle nuclei**
**TJP3**	**pyd**	**7**	**33386**	**Polychaetoid; cell adhesion molecule binding; scaffolding**
**TTN**	**bt**	**9**	**31545**	**Projectin; associated with myosin thick filaments**

Several of the human genes shared a common *Drosophila* orthologue, for example, *ACSM1* and *ACSM2* (*pdgy*); *SYNE1* and *SYNE2* (*msp300*) and *CHIT1* and *OVGP1* (*cht7*). TRiP RNAi lines for 2 genes (*shn*, *unc-89*) were unhealthy, and experimental crosses were unsuccessful. For *Drosophila Papss* (human orthologue *PAPSS1*), no adult survivors of the correct genotype were found after crosses to 2 different TRiP RNAi lines.

**Fig 2:**
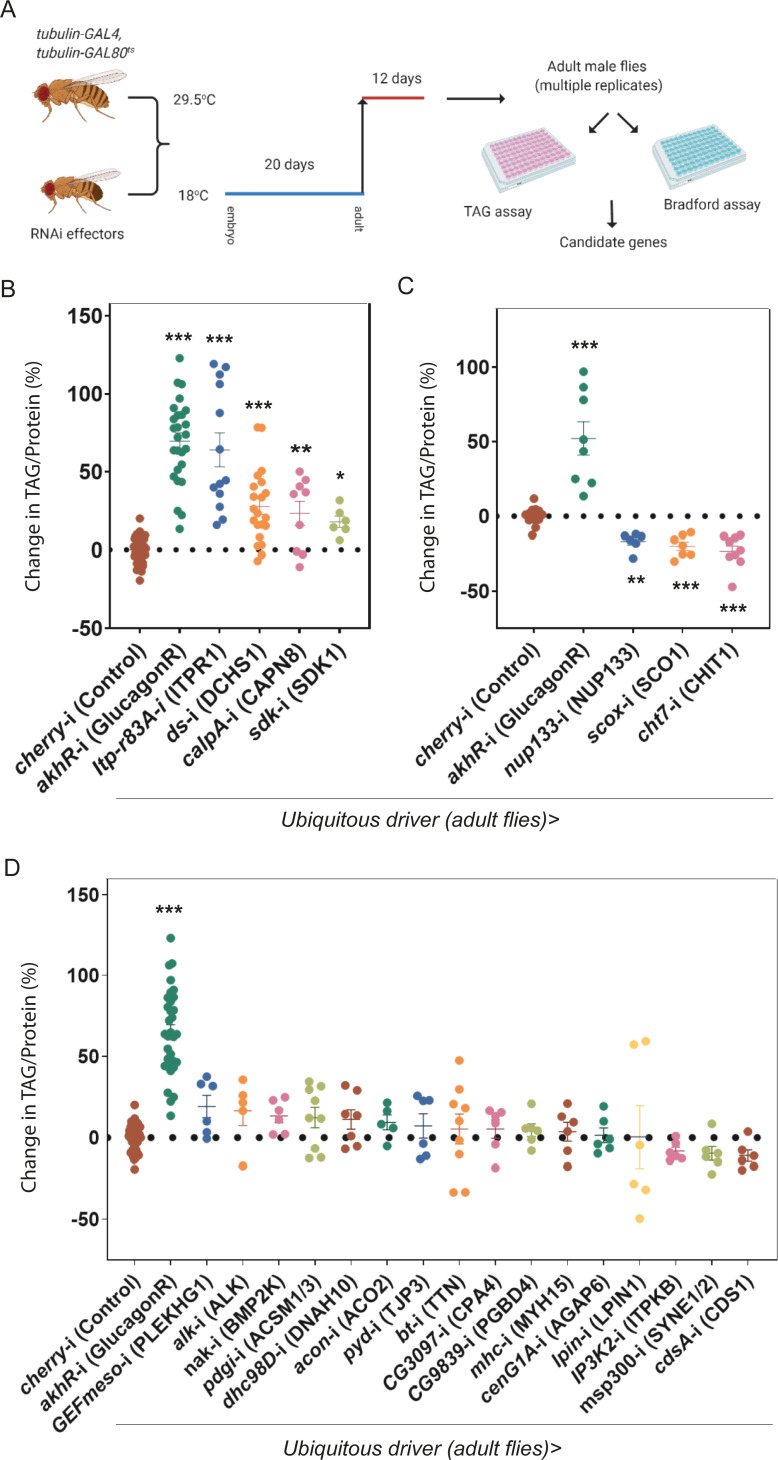
Experimental design of the *Drosophila* screen to identify novel obesity genes. **(A)** Schematic of the functional screen in *Drosophila*. **(B, C, D)** TAG levels normalised to level of protein upon ubiquitous adult specific knockdown of *Drosophila* orthologues of human genes from severely obese people. Each data point corresponds to an average of 3 technical replicates of TAG normalised to protein levels obtained from 10 male flies. Results obtained from multiple replicates are shown for knockdown of each gene. Error bars represent SEM; *p < 0.05, **p < 0.01 and ***p < 0.001 versus control by Mann–Whitney *U* Test. The underlying data for this figure can be found in S7 Table. Fig 2A was “created with BioRender.com.” TAG, triacylglyceride.

For each of the genes selected for further study, we knocked down expression in a spatially and temporally controlled fashion using the GAL4 system [21] with GAL80^ts^ [22,23]. Using this system, GAL4-driven transgenes (UAS-RNAi) are expressed at the restrictive temperature for GAL80^ts^, 29.5°C, but not at the permissive temperature, 18°C, at which GAL80^ts^ is active and represses GAL4. Lines from the TRiP RNAi collection targeting each of the 24 genes were crossed to *tubulin-GAL4*, *tubulin-GAL80*^*ts*^ to drive expression ubiquitously in adults. So as not to inhibit expression of the target gene during embryonic and larval development, the parents of each cross and their progeny were kept at 18°C until eclosion (**Fig 2A**). The adults were then shifted to 29.5°C for 12 days to induce RNAi expression. Adult males were frozen in batches of 10 for further analysis.

### Identification of candidate obesity genes

To assay increased adiposity in adults, we measured levels of TAG, the major storage form of fat in the body [24] using a coupled colorimetric assay normalised to levels of protein [25] (**Fig 2A**). As a positive control, we measured TAG levels after ubiquitous knockdown of the *Drosophila* glucagon receptor orthologue (*adipokinetic hormone receptor* (*akhR*)) [26] (**Fig 2B–2D**). Loss of function of *akhR* has been shown to increase levels of TAG [26,27], and we observed a dramatic increase in TAG levels (**Fig 2B–2D**).

For each knockdown experiment, we performed between 6 and 21 biological replicates (10 male flies per replicate). TAG and Bradford assays were carried out in 96-well plates with each plate having 3 or more biological replicates (as well as 3 technical replicates for each biological replicate) of control RNAi (cherry), *akhR* knockdown, and experimental samples (**Fig 2A**).

We found significant increases in TAG normalised to protein levels after knockdown of 4 out of 24 genes (*itpr*, *dachsous*, *calpA*, and *sdk*; **Fig 2B**). *itpr* encodes an intracellular ligand–gated calcium channel, Inositol 1,4,5 trisphosphate receptor (IP3R). Whereas the loss of IP3R during early development is lethal, hypomorphic mutations or targeted knockdown in neuropeptide secreting neurons demonstrated a role for *itpr* in obesity [18,28,29] in line with our results. Down-regulating *calpA*, a calcium-dependent endopeptidase orthologous to human *CAPN8*, also increased levels of TAG. *calpA* is expressed in the adult midgut endocrine cells [30] and mammalian *CAPN8* in the gastrointestinal mucous secreting cells [31], suggesting possible metabolic roles for both orthologues. A third gene, *sidekick* (*sdk*; *SDK1* in humans), a fibronectin family member [32,33] also raised TAG levels after knockdown. No role in regulating metabolism or obesity has been discovered previously for either *calpA*/*CAPN8* or *sdk/SDK1*.

**Fig 3:**
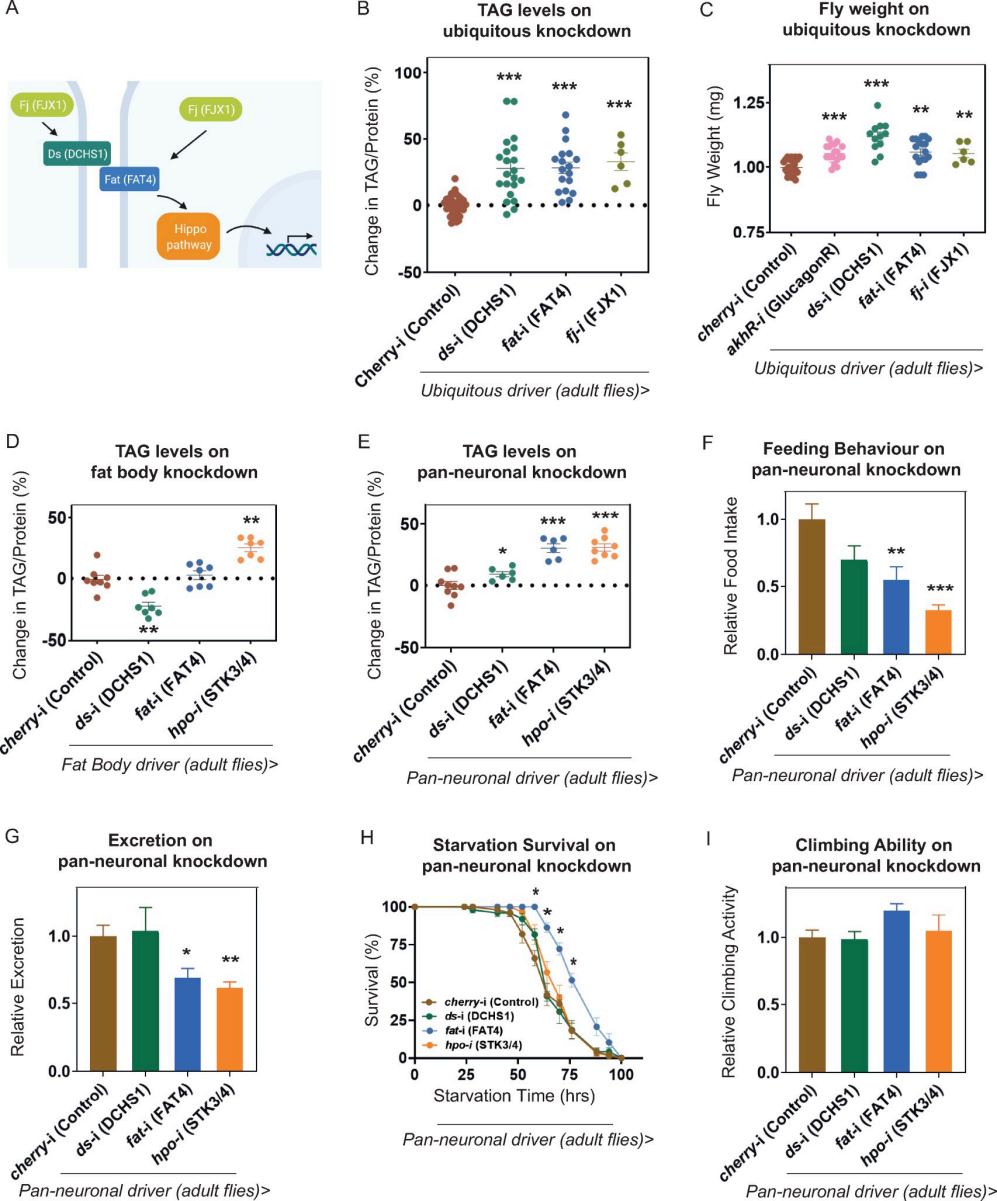
Dachsous, Fat, Four-jointed, and Hippo regulate *Drosophila* obesity. (A) A schematic of Dachsous and Fat signalling pathways. (B) TAG levels normalised to level of protein upon ubiquitous knockdown of *Drosophila dachsous*, *fat*, or *fj* in adult flies. Each data point corresponds to an average of 3 technical replicates of TAG normalised to protein levels obtained from 10 male flies. Results obtained from multiple replicates are shown for knockdown of both genes. (C) Fly weight upon ubiquitous knockdown of *Drosophila dachsous*, *fat*, or *fj*. Each data point corresponds to the weight of 8 to 10 males normalised to number of flies. TAG levels normalised to level of protein in adult male flies upon adult specific (D) fat body and (E) pan-neuronal knockdown of *dachsous*, *fat*, *and hpo*. Adult-specific pan-neuronal knockdown of *ds*, *fat*, or *hpo* decreases food intake and excretion on *fat* and *hpo* knockdown (F, G), increases survival upon starvation after *fat* knockdown (H), and does not affect climbing ability (I). Results obtained from multiple replicates are shown for all genes. Error bars represent SEM; *p < 0.05, **p < 0.01 and ***p < 0.001 versus control by Mann–Whitney *U* Test. The underlying data for this figure can be found in S7 Table. Fig 3A was “created with BioRender.com.” TAG, triacylglyceride.

Knockdown of *dachsous* (*ds*, human *DCHS1*) also resulted in obese flies (**Fig 2B**). Dachsous is a member of the cadherin family of transmembrane receptors [34] and is known to play a role in 2 well-studied signalling pathways, the Hippo signalling pathway (**Fig 3A**) and the planar cell polarity pathway [35]. Dachsous mediates many of its actions, both in *Drosophila* and in mammals, through binding to another cadherin family protein, called Fat [36]. As of yet, no role for Dachsous in regulating metabolism or obesity has been reported in any organism.

Interestingly, we also found 3 genes that, when down-regulated, gave leaner, rather than fatter, flies as evidenced by decreased TAG levels (*nup133*, *Scox*, and *cht7*; **Fig 2C**). The first encodes the nucleoporin, *nup133*, which belongs to the Y-complex (also known as Nup107-160 complex), an important component of the nuclear pore complex scaffold [37]. Although it seems surprising that mutations in a nuclear pore protein, which is required in most if not all cell types, would be involved specifically in the regulation of body fat, mutations in a number of nuclear pore components have been linked to human hereditary diseases [38]. The second gene, *Scox* (human *SCO1*), encodes a copper chaperone involved in the synthesis of cytochrome C oxidase in mitochondria. *Scox* mutant larvae were found to be growth defective [39], a phenotype comparable to what we observe after *Scox* knockdown. Down-regulation of the third gene, *Chitinase 7* (*Cht7*; human *CHIT1*), also resulted in flies with less TAG. Knockdown of the remaining 16 genes did not result in a significant difference in TAG levels (**Fig 2D**).

### Role of Dachsous, Fat, and Hippo pathway

Of the genes that increased TAG levels, we focused further attention on the cadherin family member, Dachsous, which has been well studied in *Drosophila* and for which interacting partners have been characterised (**Fig 3A**). The extracellular domain of Dachsous interacts with the transmembrane protein Fat [35]. Initially identified almost 100 years ago, *fat* mutants were so-named due to their “short, thick thorax and abdomen” [40–42]. In spite of its mutant phenotype, a role for *fat* in obesity has not been reported previously. To test whether Fat, and any of the regulatory pathways in which Dachsous and Fat interact, regulates obesity we knocked down expression of *fat* in adults by RNAi. Promisingly, we found that knockdown of *fat* also increased TAG levels (**Fig 3B**).

The interaction between Dachsous and Fat is regulated by phosphorylation in the Golgi of their extracellular cadherin repeats by a third protein, Four-jointed (Fj) [43,44] (**Fig 3A**). We found that ubiquitous knockdown of *fj* also increased TAG levels (**Fig 3B**). Knockdown of *ds*, *fat*, or *fj* led to an increase in body weight of the adult male flies (**Fig 3C**). Our results suggest that Dachsous, Fat, and Four-jointed together regulate one or more pathways, which, when disrupted, lead to obesity.

In *Drosophila*, Fat and Dachsous regulate the evolutionarily conserved Hippo signalling pathway, a key pathway in the control of cell proliferation and organ size [35,45]. Initially discovered in *Drosophila*, the core of the Hippo pathway consists of the kinases Hippo (MST 1/2 in mammals) and Warts (LATS 1/2), which block nuclear entry of the transcriptional coactivator Yorkie (YAP/TAZ) [46]. Interestingly, in *Drosophila*, Hippo signalling has been found to regulate fat accumulation in the fat body, an organ analogous to the human adipose tissue and liver [47]. Therefore, we hypothesised that perturbation of the Hippo signalling pathway would result in obese flies.

To test this, we down-regulated the Hippo pathway by ubiquitous knockdown of the *hpo* gene or by expression of the active form of *yki* (*yki*^*act*^) [48] in adults, using the conditional *tubGAL4;tubGAL80*^*ts*^ system. After expression of *hpo*-RNAi or *yki*^*act*^ (**S1A–S1C Fig**), we found that TAG levels were dramatically reduced, rather than increased, when compared to controls (**S1D Fig**). This was accompanied by significant degeneration of internal organs (**S1C Fig**) and a reduction in protein levels (**S1E FIg**). These phenotypes have previously been reported after intestinal expression of *yki*^*act*^ and are due to cachexia (organ wasting) [49]. As we did not observe cachexia-like organ wasting after knockdown of Dachsous or Fat, we infer that they do not function upstream of Hippo signalling in the context of cachexia.

### Dachsous, Fat, and Hippo act in the nervous system to regulate obesity

Whereas the loss of Hippo signalling throughout the organism resulted in cachexia, down-regulation of Hippo in the fat body alone has been shown to increase body fat [47]. Therefore, we tested whether tissue-specific knockdown of *ds* or *fat* would lead to obesity. We knocked down *ds*, *fat*, or *hippo* specifically in the fat body (*lpp-GAL4*) in adults and assessed TAG levels. Encouragingly, fat body–specific knockdown of *hippo* increased TAG levels (**Fig 3D**) as previously reported [47]. However, we observed no significant change in TAG levels after fat body–specific knockdown of *fat*. Furthermore, knockdown of *ds*, surprisingly, led to decreased TAG (**Fig 3D**). Together, our results suggest that, in the context of increased adiposity, signalling through *ds/fat* is not coupled to the Hippo pathway in the fat body.

The brain plays a central role in integrating external and internal stimuli to maintain energy homeostasis. For example, components of the leptin and melanocortin signalling pathways function in the brain to regulate energy balance [2]. Furthermore, the brain plays a critical role in regulating fat storage in *Drosophila* [17,50,51]. Therefore, we knocked down *ds*, *fat*, or *hippo* specifically in the nervous system of adult flies (*elaV-GAL4*) and assessed adiposity. We observed significant increases in TAG levels upon knockdown of *hippo* or *fat* and a small but significant increase after knockdown of *ds* (**Fig 3E**). We conclude, therefore, that Hippo pathway activity in the nervous system regulates adiposity.

We examined the physiological basis of the obesity phenotype observed after adult-specific neuronal knockdown of *ds*, *fat*, or *hpo* by assessing 4 physiological parameters: feeding behaviour, excretion, starvation resistance, and locomotive ability. First, feeding assays were carried out using the “Capillary Feeder assay” (CAFÉ assay) [52]. Surprisingly, we found that *fat* or *hpo* knockdown significantly decreased food intake while there is a smaller reduction on *ds* knockdown (Fig 3F). Interestingly, these results parallel findings from studies on *Akh*- and *AkhR*-deficient flies, where obesity and increased TAG levels were accompanied by decreased food intake [53]. Second, we carried out CAFÉ-excretion studies [54] and found reduced excretion upon *fat* or *hpo* knockdown and no significant difference on *ds* knockdown, complementing our observations with feeding behaviour (Fig 3G).

Third, starvation survival assays, where flies were food deprived (water only) to assess their capacity to mobilise energy reserves. We found that neuronal knockdown of *fat* led to significantly increased starvation resistance as compared to controls, as might be expected from increased TAG levels (Fig 3H). However, no significant change in starvation survival was seen after *ds* or *hpo* knockdown. A more detailed assessment of metabolic parameters would be required to gain further insights into this observation.

Fourth, we assessed locomotive ability with a climbing assay that makes use of the negative geotaxis behaviour of flies and provides a quantitative measure [55]. Adult-specific neuronal knockdown of *ds*, *fat*, or *hpo* did not significantly alter climbing ability (Fig 3I) demonstrating that general locomotion is not perturbed.

### Variants in human orthologues of genes identified in the *Drosophila* screen

**Fig 4:**
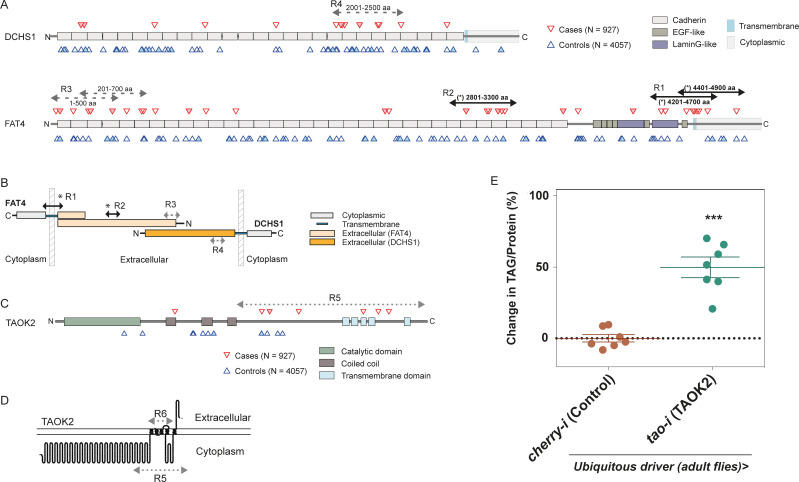
Rare variants in *DCHS1*, *FAT4*, and the Hippo pathway in severely obese people and healthy controls. (A) Locations of very rare (MAF < 0.025%) nonsynonymous variants in DCHS1 or FAT4. Variants are shown in severely obese cases (*n =* 927 people; red, upper triangles) or INTERVAL controls (*n* = 4,057 people; blue, lower triangles). Filled triangles indicate VEP IMPACT = HIGH or SNV predicted damaging. A sliding window approach was used to perform burden tests (SKATBinary, method = “burden”) within localised regions (window size = 500 aa, shift = 200 aa); asterisk (*, black, R1-R2) indicates windows with burden test *p*-value < 0.05 after BH adjustment for multiple sliding windows; grey (R3-R4), nominal *p*-value < 0.05. (B) Cartoon illustrating DCHS1-FAT4 and the location of regions R1-R4, as (A). (C, D) TAOK2 sliding window analysis of very rare variants (MAF < 0.025%) **(S4 Table)**. (E) TAG levels normalised to level of protein in adult male flies upon adult specific ubiquitous knockdown of *Drosophila tao*. Error bars represent SEM; ***p < 0.001 versus control by Mann–Whitney *U* Test. The underlying data for this figure can be found in S7 Table. aa, amino acid; BH, Benjamini–Hochberg; MAF, minor allele frequency; SNV, single-nucleotide variant; TAG, triacylglyceride.

To test whether rare variants in the human counterparts of Dachsous, Fat, Four-jointed, and Hippo, or their signalling pathways, contribute to severe human obesity, we performed exploratory analysis using WES data available on 927 severely obese individuals from the GOOS cohort who are of UK Caucasian ancestry from the GOOS cohort (referred to as the SCOOP cohort in previous studies) [56,57] and 4,057 healthy volunteers (INTERVAL cohort) (**S3 Table)**. We first examined *DCHS1* and *FAT4* for gene-based burden of rare (MAF < 0.1%) and very rare (MAF < 0.025%) variants. As whole-gene analysis can mask effects that may be constrained to regions within a gene, we also performed an additional exploratory analysis of very rare variants in localised coding regions using overlapping windows of width 1,500 bp (sliding window analysis; **Methods**). Two regions in FAT4 reached Benjamini–Hochberg (BH)-adjusted *p* < 0.05 (adjusting for multiple sliding windows within the gene) with an odds ratio (OR) > 1 **(Fig 4A, S4 Table)**. These regions encompass the FAT4 transmembrane domain and cadherin repeats 28 to 30 (**Fig 4A and 4B**, regions R1 to R2). Further work will be needed to assess whether these genetic findings replicate in other cohorts.

We identified a set of genes encoding further components of the human Hippo signalling pathway and performed gene-based analysis of very rare (MAF < 0.025%) and rare (MAF < 0.1%) variants in severely obese versus controls **(S3 Table)**. While we are underpowered to formally test for enrichment of rare variants in any single gene in this set, we observed nominal significance for the burden of rare variants in *TAOK2* (**S3 Table**). Exploratory sliding window analysis of very rare variants across the gene set also indicated a region of *TAOK2* **(Fig 4C and 4D, S4 Table)**. Although the expected number of variants in obese cases was small, we hypothesise that a region of *TAOK2* encompassing the transmembrane domains may be enriched for very rare variants in severely obese cases **(Fig 4C and 4D)**.

To test these hypotheses in an independent cohort, we interrogated very rare variants (MAF < 0.025%) in *TAOK2*, *DCHS1*, and *FAT4* in approximately 50,000 exomes from UK Biobank (**Methods**) using a case–control approach for severe obesity (*n* = 925 cases, BMI > 40 kg/m^2^; *n* = 46,673 controls, BMI ≤ 40 kg/m^2^). We did not find an association of rare variants in *DCHS1* or *FAT4*, nor in the specific regions of *DCHS1* or *FAT4* that we interrogated, with severe obesity in UK Biobank **(S5 Table**). These observations will require further study in larger cohorts.

We did find a nominally significant burden of very rare coding variants in *TAOK2* in people with severe obesity (*n* = 225 markers, *p* = 0.001, SKAT-O test; #case carriers = 14, #control carriers = 426, OR = 1.6 (0.9 to 2.8), *p* = 0.08, Fisher’s exact test) **(S5 Table, S2 Fig).** Analysis of the 2 specified regions of *TAOK2* also indicated an association of rare variants in these regions with severe obesity **(S5 Table, S2 Fig)**. Interestingly, *TAOK2* is located on human chromosome 16p11.2, a region in which deletions are associated with neurodevelopmental disorders and obesity [58,59].

The *Drosophila* orthologue of *TAOK2*, Tao, is a Sterile 20 family Serine/Threonine kinase that directly phosphorylates and activates Hippo [60,61]. Given the association between rare variants in *TAOK2* and severe obesity, we assessed the functional significance of this finding by knockdown of *tao* expression in *Drosophila*. We found that ubiquitous knockdown in adults significantly increased TAG levels (**Fig 4E**), further supporting the role of Hippo signalling in regulating adiposity.

## Discussion

Here, we demonstrate the success of functional screens in *Drosophila* in assessing the likely pathogenicity of rare variants in human genes associated with severe obesity and in predicting novel candidate human obesity genes and signalling pathways. Obesity is a systems-level disorder arising from complex interactions between multiple organ systems. An in-depth understanding of obesity benefits from studies in simple model organisms where this integrative network can be comprehensively examined in vivo. The use of *Drosophila* as a model system enables the investigation of genetic pathways underlying obesity at a whole organism level. This work provides a template for identifying genes carrying pathogenic variants relatively rapidly and for investigating the signalling pathways within which these proteins act. In this way, the identification of candidate obesity causing genes from severely obese people has the potential to uncover entire signalling pathways linked to obesity.

We identified homozygous coding variants in a large number of genes by studying people with severe obesity including those from consanguineous families. From 61 genes studied, we identified 7 genes that, when knocked down in *Drosophila*, lead to changes in whole body TAG levels: *itpr*, *calpA*, *sdk*, *ds*, *nup133*, *scox*, and *cht7*. Six of the 7 genes had not been shown previously to regulate adiposity in either *Drosophila* or humans.

Using an inducible RNAi system for gene knockdown, we were able to avoid developmental defects that might be associated with a reduction in gene activity. This proved to be significant, as we found that unrestricted knockdown of *ds* resulted in early lethality. From our results, we inferred that mutations in 4 of the human genes (*IP3R*, *CAPN8*, *SDK1*, and *DCHS1*) were likely to be loss of function or hypomorphic variants, as knockdown of orthologous genes in *Drosophila* raised TAG levels. Based on the reduction in TAG observed on knockdown of *nup133*, *scox*, and *cht7*, we hypothesise that the human variants in these genes may be gain-of-function mutations. This could be tested in *Drosophila* by overexpressing the wild-type genes or by introducing the mutations found in the human variants into the corresponding *Drosophila* genes.

Knockdown of *ds*, a member of the Hippo signalling pathway, resulted in obesity prompting us to investigate other proteins acting in the Hippo pathway. This proved fruitful as we were able to identify 3 further genes controlling adiposity in *Drosophila*: *fat*, *fj*, and *hippo*. We found that neuronal knockdown of *ds*, *fat*, and *hippo* led to increased TAG levels in adults. Previous genome-wide association studies of BMI in humans have provided strong evidence for a role of the nervous system in obesity susceptibility in the population [62] as well as in monogenic obesity. Identifying the specific neurons in which the Hippo pathway acts will help to reveal the neuronal circuitry regulating adiposity.

Surprisingly, and contrary to expectation, we observed a decrease in food intake after knockdown of *fat*, *hpo*, or *ds*. This is similar to observations of *Akh*- and *AkhR*-deficient flies, where increased TAG levels are accompanied by decreased food intake [53]. Although the physiological basis for this hypophagia is not known, the study found that *Akh* mutants have increased expression of the orexigenic genes *NPF* and *CChamide-2*. This could be a compensatory homeostatic increase but suggests that the effect of *Akh* on feeding is independent, or downstream of, these orexigenic peptides. Additionally, AKH signalling was found to regulate expression of other metabolic genes such as Corazonin, Limostatin, and Insulin-like peptides [53]. Similar mechanisms might be operating in the case of *ds*, *fat*, and *hippo* and will require further investigation.

Our study serves as a proof of principle that *Drosophila* functional screens are an efficient and effective way to assess the likely pathogenicity of rare exonic variants associated with human obesity. With this approach, we were not only able to identify 7 genes most likely to regulate human adiposity, we were also able to predict further novel candidate human obesity genes, for example, *TAOK2*, which we then showed to be functionally relevant as a regulator of obesity in *Drosophila*. Future work to generate precision obesity models incorporating patient-specific genetic mutations using CRISPR/Cas9 technology will elucidate further the role of the variants and their associated genes in the regulation of obesity.

## Methods

### Human studies

All human studies were approved by the Cambridge Local Research Ethics Committee (03/103), and all participants and their parents (for children below the age of 16) gave written informed consent. All research was conducted in line with the principles outlined in the Declaration of Helsinki.

### Whole-exome sequencing and variant annotation for autosomal homozygous variants

Exome sequencing and variant-calling was performed in 2 batches (Beijing Genomics Institute, BGI). For 50 patients, BGI performed exome sequencing (Agilent 51M exon capture kit), hg19 alignment, variant-calling (UnifiedGenotyper), and variant quality filtering based on GATK Best Practices [63]; variants were excluded if read-depth DP < 4 or (MQ0 ≥ 4 and MQ0/DP > 0.1); sample coverage (%target at read-depth ≥20×: median of samples = 92.4%, min = 89.4%; ≥4×: median = 98.9%, min = 97.6%). For 23 patients, BGI performed exomes sequencing (SureSelect All Exon 38M exon capture kit) at lower coverage (%target at read-depth ≥20×: approximately 60%; ≥4×: approximately 93%); reads were then aligned to hg19 followed by variant-calling (HaplotypeCaller) and hard-threshold variant quality filtering according to GATK v3.7 Best Practices [63], and variant-level quality control was performed using hard-threshold filtering to retain SNVs (QD ≥ 2, MQ ≥ 40, FS ≤ 60, SOR ≤ 3, MQRankSum ≥ −12.5, ReadPosRankSum ≥ −8) and indels (QD ≥ 2, ReadPosRankSum ≥ −20, InbreedingCoeff ≥ −0.8, FS ≤ 200, SOR ≤ 10). Runs-of-homozygosity analysis was performed from bam files using H3M2 [64] with default parameters. Variants were filtered to retain homozygous variants using SelectVariants (isHOMVar) from GATK v3.7, annotated for gnomAD exomes [65] using Annovar (version 1 February 2016; hg19_gnomad_exome, version 11 March 2017) and filtered to retain homozygous variants with MAF <1% in gnomAD exomes (gnomAD_exome_ALL < 0.01 or missing). The resulting autosomal homozygous variants were annotated using Ensembl VEP v90 [66] with respect to Ensembl canonical transcripts and filtered to retain nonsynonymous SNVs, splice region variants, and indels. Variants with MAF <1% among all exomes available in gnomAD and <1% in the gnomAD South Asian subpopulation were retained. Further filtering of variants against existing and emerging population sequencing datasets will be required to facilitate prioritisation of variants for further studies. After these variant filtering steps, genes were taken forward for homology analysis in *Drosophila* if they contained a variant annotated as VEP “Impact = HIGH” (resulting in VEP “Consequence” of “stop-gain,” “frameshift_insertion” or “frameshift_deletion” in this variant set), or if at least 2 affected individuals carried a nonsynonymous SNV or “Impact = HIGH” variant.

### Rare variants in SCOOP-INTERVAL exomes

Whole-exome variant calls from the SCOOP and INTERVAL cohorts were obtained from the UK10K-INTERVAL study as previously described [67]. Details of sequencing and variant-calling can be found elsewhere [57]. *N =* 927 SCOOP cases and *n =* 4,057 INTERVAL controls survived QC as previously described [57] and were used in this study.

For this study, SCOOP-INTERVAL variants were annotated using Ensembl VEP v96 [66], and population frequencies for additional population studies were obtained using Annovar (version 16 April 2018; hg19; gnomad_genome, gnomad211_exome, gnomad211_genome, popfreq_all_20150413, abraom, gme, hrcr1, kaviar_20150923) [68]. Variants were defined as rare (MAF < 0.1%) or very rare (MAF < 0.025%) if the allele frequency thresholds at that position were satisfied in every queried population and among the SCOOP-INTERVAL samples used in this study. Exonic nonsynonymous or splice region variants were defined using VEP filter “(Impact in HIGH,MODERATE) or (Consequence is splice_region_variant)” with respect to the Ensembl canonical transcript. We defined a further nested category of variants summarised as “high impact or predicted damaging,” defined as variants with Ensembl VEP impact = HIGH (including stop-gains, stop-loss, and frameshift variants) and SNVs predicted to be damaging by both SIFT and PolyPhen2 (SIFT “deleterious” and PolyPhen2 “probably_damaging”).

Gene-based and sliding window analysis was performed for a selected list of genes for further study in SCOOP-INTERVAL exomes. The selected genes comprised components of the Hippo pathway and the genes in our TAG *Drosophila* screen.

Gene-based analysis of rare (MAF < 0.1%) or very rare (MAF < 0.025%) exonic nonsynonymous or splice region variants in SCOOP-INTERVAL were performed using *SKATBinary* (method = “burden” or “SKAT-O”, method.bin = “UA”) from R package SKAT (v2.0.0 (Lee and colleagues, 2012; SKAT v2 package [69]. *P* values are reported before and after adjustment for multiple tests (genes) using Benjamini–Hochberg (BH) and Holm methods.

Sliding window analysis of very rare (MAF < 0.025%) exonic nonsynonymous variants in SCOOP-INTERVAL were performed in overlapping windows of the Ensembl canonical transcript using custom scripts. Window size was 1,500 bp (corresponding to 500 aa), and sliding windows were overlapped by 600 bp (200 aa). Variants were included in the analysis if the VEP annotated “Protein_position” was located within the window. Burden tests within sliding windows were performed using *SKATBinary* (method = “burden”, method.bin = “UA”) as for the gene-based analysis described above. Multiple testing corrections (BH and Holm methods) are reported with respect to all sliding windows in the selected gene list (approximately 3,200 windows in total).

### Rare variants in UK Biobank 50k FE exomes and severe obesity

This research was conducted using the UK Biobank Resource under Application Number 53821. We used the UK Biobank PLINK-formatted variant files from the FE exome pipeline (UK Biobank Field 23160; *n* = 49,960 individuals) [70]. Variant annotation was performed using Annovar (version 16 April 2018; hg38; refGene, ensGene, gnomad211_exome) [68] and filtered to retain nonsynonymous exonic or splicing variants. Variant filtering for population MAF was based on the annotation field “AF_popmax.” Relatedness was obtained from the UK Biobank Genetic Data resource (ukbgene rel). BMI (kg/m^2^) was obtained from the UK Biobank initial assessment visit (UK Biobank Field 21001, Instance 0), and this value was available to us for *n* = 47,599/47,766 unrelated individuals with FE exomes (kinship < 0.0442). Because this study was focused only on very rare variants and severe obesity (BMI > 40 kg/m^2^), we included all unrelated individuals with available BMI regardless of age, sex, self-reported ethnicity, reported genetic ethnicity, and genetic principal components of common variation and did not use as covariates in formal burden tests. Fisher’s exact tests were performed for cases (severely obese, BMI > 40 kg/m^2^) for versus controls (BMI < = 40 kg/m^2^). Burden tests and SKAT-O tests for cases and controls were performed as described above using variants resulting in an amino acid change in RefSeq transcripts NM_003737 (*DCHS1*), NM_024582 (*FAT4*) or NM_016151 (*TAOK2*).

### Homozygous rare variants in Genes & Health study cohorts

Variant calls (VCFs) from exome sequencing of 8,000 people from the Genes & Health study were downloaded from the European Genome-phenome Archive at the European Bioinformatics Institute (Study ID EGAS00001001565, Dataset ID EGAD00001004581). Approximately 3,781 people were from the East London Genes & Health (ELGH) cohort [71] (Bangladeshi and Pakistani, with self-stated related parents); 2,791 people from the Born in Bradford cohort (Pakistani, mostly self-stated or DNA autozygous individuals); and 1,428 people from the Birmingham cohort (Pakistani, unselected) [7]. About 86/8,086 samples in the downloaded VCFs originated from a rare disease study and were excluded. Variants were filtered to retain SNPs and indels with {PASS, FS < 30, GQ > 10}. The provided variant annotation (Ensembl VEP v85, hg38) was used to filter variants to retain homozygous exonic nonsynonymous or splice donor/acceptor variants (VEP Impact in “HIGH,MODERATE” with respect to the Ensembl canonical transcript). Population frequency thresholds were defined with respect to the provided annotation for 1000G and ExAC subpopulations and the allele frequency among ELGH samples inspected in this study (**S6 Table**).

### *Drosophila* stocks and media

Transgenic RNAi lines (Harvard TRiP library; www.flyrnai.org), *UAS- yki*^*act*^ and *elaV- GAL4* were obtained from BDSC, *itpr* RNAi strain (1063R-2) from the National Institute of Genetics, Kyoto, Japan. *tubulin-GAL4*, *tubulin-GAL80*^*ts*^ driver line was a gift from Dr Timothy Megraw. *lpp-GAL4* line was a gift from Dr Suzanne Eaton. Male transgenic RNAi flies were crossed to either *tubulin-GAL4*, *tubulin-GAL80*^*ts*^ or *tubulin-GAL80*^*ts*^*; elaV- GAL4* or *tubulin-GAL80*^*ts*^*; lpp- GAL4* virgin females. The progeny were allowed to develop at 18°C until eclosion of adult flies. Adults were then collected and transferred to 29.5°C for 12 days with the flies transferred to fresh food every 2 to 3 days. Males of the correct genotype were then collected in batches of 10, weighed and flash frozen and stored at −80°C for further analysis.

Flies were reared on standard fly food containing, per litre, 7.5 g agar (Oxoid agar number 2; LP0012), 55 g glucose (Fischer chemicals; CAS 50-99-7), 50 g dry yeast (Kerry; 20050488), 35 g wheat flour (Cann Mills Stoneground), 25 ml of 10% Nipagin (Chemlink Speciality; CLA-CHIGINM), 4 ml Propionic acid (Merck; 79-09-4), and 10 ml Penicillin/Streptomycin (GIBCO; 15140–122).

### TAG analysis

The TAG assay was adapted from [72]. Multiple batches of 10 male flies each were processed for TAG analysis. A small scoop of zirconium beads and 300 μl of PBS, 0.05% Tween was added to each tube. The samples were then homogenised using a Precellys homogeniser. For protein estimation, 40 μl of homogenised sample was immediately collected and frozen. The remaining sample was heat inactivated for 10 minutes at 70°C. A volume of 200 μl of the heat-inactivated sample was transferred to fresh tubes. A volume of 4 μl of lipase (25 KU/mL; Merck; 437707) was added to each tube and mixed. The samples were then kept at 37°C overnight. After overnight incubation, the samples were centrifuged at 14,000 rpm for 3 minutes, and the supernatant collected. Each sample was then put in triplicates in a 96-well plate. Different concentrations of glycerol were used as standards. Prewarmed Free Glycerol reagent (Merck; F6428) was then added to the 96-well plate, and the plate was incubated at 37°C for 6 minutes. After a brief spin, absorbance measurements were taken at 540 nm using a Hidex Sense Plate Reader. The Bradford assay was used for protein estimation. Briefly, pre-heat-inactivated sample was centrifuged, and the supernatant put in triplicates in a 96-well plate. Bradford reagent was then added, and absorbance measurements taken at 600 nm.

### Physiological assays

For all assays, male transgenic RNAi flies were crossed to *tubulin-GAL80*^*ts*^*; elaV- GAL4* virgin females. The progeny were allowed to develop at 18°C until eclosion of adult flies. Adults were then collected and transferred to 29.5°C for 12 days with the flies transferred to fresh food every 2 to 3 days. Males of the correct genotype were then collected in batches of around 10 flies each for the assays.

Food intake was quantified using a modified CAFÉ assay [52] carried out at 29.5°C. CAFÉ chambers were made from empty standard fly vials and fly plugs soaked in water (to maintain CAFÉ chamber humidity and serve as a source of water). A 5-μl capillary micropipette (Hirschmann) containing liquid food (2.5% sucrose, 2.5% inactivated yeast, and 0.5% blue food dye (FD&C Blue 1; Merck) was inserted through the fly plug in each chamber. Flies were left overnight for habituation with a capillary of liquid food (without blue dye). The capillary was replaced with liquid food (with blue dye), and food consumption was recorded after 6 hours. Excretion studies were carried out using CAFÉ-excretion assays [54] with fresh capillaries containing liquid food (without blue dye) inserted into the same CAFÉ chambers for 18 hours. The excreted blue waste that accumulated on the vials was then collected by adding 1 ml of water to each vial and absorbance measured at 630 nm. For each cross, 10 biological replicates (10 male flies per replicate) were assessed.

Starvation survival assays were carried out at 29.5°C by keeping male flies in empty standard fly vials. Fly plugs soaked in water provided a source of water and maintained humidity and were hydrated frequently. The number of dead flies was scored every 6 to 10 hours. For each cross, 3 to 6 biological replicates (10 male flies per replicate) were assessed.

Climbing assays to assess for locomotion defects are based on the negative geotaxis behaviour of flies [55]. These were carried out using 6 empty standard fly vials that were stuck next to each other on a flat ruler. Male flies in batches of around 10 were transferred into each vial, and another empty vial was then sealed over. The fly vials were tapped together to displace the flies to the bottom of the vial, and video recordings were made of their climbing behaviour. The number of flies crossing a 5-cm mark on the bottom vial in 10 seconds was counted. Multiple trials were conducted for each batch with 1 minute of recovery time in between. Climbing activity was determined by dividing the average number of flies that crossed the target line by the total number of flies in that batch.

## Supporting information

S1 Fig Ubiquitous knockdown of Hippo signalling leads to cachexia. (A, B) Expression of *hpo*-RNAi or *yki*^*act*^ results in flies with a “bloated” abdomen. (C) Dissection of the bloated flies revealed significant degeneration of internal organs as compared to controls. (D) TAG levels normalised to level of protein and (E) protein levels upon ubiquitous knockdown of *hpo* or misexpression of activated *yki* in adult flies. Each data point corresponds to an average of 3 technical replicates of TAG normalised to protein levels obtained from 10 male flies. Results obtained from multiple replicates are shown for all genes. Error bars represent SEM; *p < 0.05, **p < 0.01, and ***p < 0.001 versus control by Mann–Whitney *U* Test. The underlying data for this figure can be found in S7 Table. TAG, triacylglyceride.
(EPS)

S2 Fig TAOK2 very rare variants (MAF < 0.025%) variants causing amino acid changes in the UK Biobank 50k FE exomes. Variants are displayed with respect to the location of the amino acid change (x-axis) versus BMI (y-axis; **Methods**). Regions R5 and R6 correspond to (**Fig 4C)**. Gene-based and region-based association tests for cases (BMI > 40) versus controls (BMI < = 40) are shown in (**S5 Table)**. BMI, body mass index; MAF, minor allele frequency.
(EPS)

S1 Table Anthropometric measurements and associated phenotypes from 73 people from a cohort with severe obesity. (XLSX)

S2 Table Genes identified in severely obese probands and their selection for the screen in *Drosophila*. (XLSX)

S3 Table Gene-level burden analysis of rare (MAF < 0.1%) or very rare (MAF < 0.025%) exonic nonsynonymous variants in Hippo signalling components using the SCOOP-INTERVAL dataset. (XLSX)

S4 Table Sliding window burden analysis of very rare (AF < 0.025%) nonsynonymous variants in SCOOP-INTERVAL. (XLSX)

S5 Table Gene-based and region analysis in UK Biobank 50k FE exomes. (XLSX)

S6 Table Summary table of rare alleles with at least 1 homozygous variant carrier. (XLSX)

S7 Table Phenotypic characterisation of human obesity candidate genes in *Drosophila*: data for Figs 2B–2D, 3B–3I, 4E, and S1D and S1E. The underlying data for S2 Fig is provided in S5 Table. The raw data for S2A and S2B Fig (UK Biobank 50K FE exomes and BMI measurements) were obtained under agreement with UK Biobank and the Project ID and Data Field IDs are in the Methods section.
(XLSX)

## Abbreviations

BH: Benjamini–Hochberg
BMI: body mass index
BMI SDS: BMI standard deviation score
CAFÉ: Capillary Feeder
DIOPT: Drosophila RNAi Screening Centre Integrative Ortholog Prediction Tool
indel: insertion/deletion
IP3R: Inositol 1,4,5 trisphosphate receptor
MAF: minor allele frequency
OR: odds ratio
SNV: single-nucleotide variant
TAG: triacylglyceride
WES: whole-exome sequencing
